# Validation of the Questionnaire “Knowledge, Attitudes and Practices” in populations of pregnant women exposed to pesticides

**DOI:** 10.1590/2317-1782/20212021285en

**Published:** 2023-06-26

**Authors:** Karlla Cassol, Cristiana Magni, Vanessa Veis Ribeiro, Maria Fernanda Capoani Garcia Mondelli, Andrea Cintra Lopes

**Affiliations:** 1 Programa de Pós-graduação em Fonoaudiologia, Faculdade de Odontologia de Bauru - FOB, Universidade de São Paulo - USP - Bauru (SP), Brasil.; 2 Universidade Estadual do Centro-Oeste - UNICENTRO - Irati (PR), Brasil.; 3 Universidade de Brasília - UnB - Brasília (DF), Brasil.

**Keywords:** Agriculture, Agrochemicals, Pregnancy, Validation Study, Clinical Protocols

## Abstract

**Purpose:**

to validate the CAP questionnaire in populations of pregnant women exposed to pesticides in the State of Paraná.

**Methods:**

382 pregnant women participated in the study, divided into two groups: Exposed to Pesticides (n = 320) and Not Exposed (n = 62). The validation process involved the validity of content, criteria and construct. The research stages were developed between August / 2018 to December / 2019 in the western and central-western regions of Paraná.

**Results:**

the instrument demonstrated an acceptable agreement on the content validity through the evaluation of judges; the criterion validity through the established criterion showed no association; in the analysis of construct validity using the technique of known groups, it demonstrated homogeneity in the variables age, nationality and family income.

**Conclusion:**

the developed analysis indicated that the psychometric properties of the validation of the Brazilian version of the scale are consistent and adequate, which allows the recommendation of the application of the instrument in a national context.

## INTRODUCTION

Brazilian agricultural production has become increasingly efficient over the years. Due to this reason, the economic sector has invested in the manufacture and sale of fertilizers and pesticides to control pests and weeds. This reality intensifies the discussions about the impacts of the indiscriminate use of pesticides and their consequences on public health^(1)^.

Data from the Brazilian Health Regulatory Agency (ANVISA) place Brazil as the largest consumer of pesticides in the world in recent harvests, with the prospect of increasing every year the intensive use of pesticides in crops^(2)^. In this ranking, Paraná is the third largest consumer from Brazil, which makes it a valid reference for research related to this theme^(3)^.

Although the discussion about the deleterious effects of pesticides on the body of directly or indirectly exposed populations is not recent, very little has been done in relation to attention and concrete intervention regarding its use. Actions aimed at minimizing the impacts of poisoning are still segregated and ineffective, making the control of the consumption and misuse of these chemical products difficult.

In recent years, studies have focused on investigating the possible harm of pesticides to farmers' health, with a focus on pregnant women, who are characterized as a higher-risk population. Research in this area points to complications and intercurrences in pregnancy, often unknown by the woman herself. This issue reveals an emerging health care concern but mainly highlights the great risk that a fragile population, such as pregnant women, is exposed to^(4)^.

The existence of any intercurrences during pregnancy can generate undesirable outcomes with different impairments to the fetus, such as low birth weight, prematurity, and congenital malformations, which are considered risk factors for infant mortality^(4,5)^. Among these factors, which put the pregnant woman's health at risk, is excessive exposure to pesticides, whose harm has already been evidenced by experimental research, which is still in progress in human beings, allowing to anticipate the presence of alterations in newborns, children of mothers who were exposed to pesticides before and during pregnancy^(4,5)^.

Although there is already evidence of the pathological consequences caused by pesticides, there are few protocols in the public health system in Brazil that efficiently and effectively investigate working women exposed to pesticides during the prenatal period. It can be inferred that this absence is based on the false idea that women, by staying at home, away from agricultural work, would not be exposed to the harmful effects of pesticides. Undoubtedly, pregnant women who live in agricultural areas or close to them are at greater risk when compared to other pregnant women, which requires greater attention and monitoring by public health policies. Not only are effective care actions necessary, but it is also urgent to establish a protocol that can place this pregnant woman in the high-risk category, and not just in the usual risk category, allowing her to have access to more specific exams that can even evaluate the levels of toxicity present in the body.

There is already a concern in other countries regarding the exposure of pregnant women to pesticides used in agriculture and in Thailand research is already being carried out using the Knowledge, Attitudes and Practices (KAP) Questionnaire, as it is an effective instrument for understanding the factors associated with exposure to pesticides in the prenatal period^(6)^.

In this sense, a translation^(7)^ of the KAP questionnaire^(6)^, which is an instrument that collaborates to identify the knowledge, attitudes, and practices of pregnant women with regard to exposure to pesticides, into Brazilian Portuguese, was carried out. In this preliminary study, carried out in Brazil, it was possible to verify significant associations between women's knowledge and the stage of pregnancy, in addition to indicating behaviors characterized as risky, as well as safe practices when dealing with toxic products. From the results obtained, it was also possible to infer that the longer the pregnancy, the greater the knowledge about the risks of exposure to pesticides, as well as the adoption of safe attitudes and practices during the gestational period, both at home and at work^(7)^.

Despite the favorable results for the use of the KAP questionnaire to survey the knowledge, attitudes, and practices of pregnant farmers in Brazil, to obtain better reliability, it is necessary to validate this instrument to verify whether it is valid for assessing these issues in a larger sample of participants.

The aim of this study was to validate the KAP questionnaire in populations of pregnant women exposed to pesticides in the State of Paraná/Brazil.

## METHODS

This research was approved by the Ethics Committee for Research Involving Human Beings of the Centro Universitário Assis Gurgacz with co-participation of the Ethics Committee for Research Involving Human Beings of the Paraná State Department of Health (CEPSH/SESA/HT) under opinion number 3.422 .972. The sample was non-probabilistic for convenience, due to the accessibility and availability of the population. Pregnant women who attended prenatal consultations and meetings at the Basic Health Units of their respective municipalities, belonging to the 4^th^ and 10^th^ Health Regions, in addition to pregnant women contacted through an active search, from August 2018 to December 2019, were recruited for this research. All individuals involved (or their guardians) signed the Free and Informed Consent Form.

The study sample included 382 pregnant women of all gestational periods, divided into two groups: Not Exposed to Pesticides - 62 pregnant women not exposed to pesticides - and Exposed to Pesticides - 320 pregnant women exposed to pesticides. The exposed pregnant women were women directly or indirectly involved in agriculture, exposed to pesticides, literate, and with apparent emotional and cognitive conditions to answer the questionnaire without help. The Not Exposed to Pesticides Group was composed of pregnant women with no direct or indirect link to agriculture. As this is the validation of an instrument, requiring a large number of subjects, the application of the instrument was carried out by Primary Health Care professionals, through training on the objectives of the instrument and its theoretical basis. The number of subjects in each test was variable.

The KAP questionnaire addresses the Knowledge, Attitudes, and Practices of pregnant farmers and includes comprehensive questions on:

- Knowledge about pesticides - information on training to use the products, routes of exposure and risks involved, acute and chronic effects on health, symptoms of toxicity, and effective methods to prevent exposure.
- Attitudes about the use of pesticides - information about beliefs for responsible and safe use, susceptibility to health effects, effectiveness of pesticides, and reason for using them.
- Practices for the safe use of pesticides - information on occupational and domestic use, use of personal protective equipment, and use of other safe precautions during and after the use of pesticides.

For validity^(8)^, which refers to the fact that an instrument measures exactly what it is intended to measure, there are three aspects that must be considered: content validity (the degree to which a test or assessment instrument evaluates all aspects to which it is designed for); criterion validity (how accurately a test measures the outcome it was designed to measure); and construct validity^(9,10)^ (whether the variables that are being tested for behave in a way to support the theory).

Regarding content validity, a committee of experts consisting of three judges, researchers in the field of toxicology that study the effects of pesticides on the human body, was formed to evaluate the instrument's items. The committee evaluated question by question using a Likert scale, assigning scores from 1 to 4 to each item presented. Shorter analysis options were used: 1 = not clear, 2 = not very clear, 3 = quite clear, 4 = very clear^(9)^. After the analysis, the items that scored 1 and 2 were excluded from the instrument, and the items that scored 3 and 4 were added and divided by the total number of responses, generating the agreement index among the specialists.

For criterion validity, as there is no gold standard instrument for correlation analysis, concurrent validity, using an established criterion, which is three brief questions that assess the same construct: “1- Do you believe you have adequate knowledge about the effects of pesticides?; 2 - Do you believe you have adequate attitudes towards pesticides?; 3 - Do you believe you have safe practices regarding pesticides?”, was chosen. The answer alternatives were yes or no. In this step, 244 pregnant women exposed to pesticides^(10)^ participated.

For construct validity, given the characteristics of the questionnaire, it was not possible to analyze the convergent validity, since the original study does not present validation tests, nor the factor analysis, since the questions that make up the questionnaire do not follow a pattern. To list the necessary evidence to guarantee construct validity, the known group´ technique was used. In this technique, the instrument was applied in two groups: Exposed to Pesticides Group, composed of 320 pregnant women, and Not Exposed to Pesticides Group, composed of 62 pregnant women^(9-11)^. Subsequently, the responses of both groups were analyzed and compared in order to verify different responses between them.

Data were analyzed using descriptive and inferential analysis. The SPSS 25.0 software was used. A descriptive analysis of nominal qualitative variables by relative frequency and percentage was performed. Descriptive analysis of discrete and continuous quantitative and ordinal qualitative variables was performed by calculating measures of central tendency (mean and median), variability (standard deviation), and position (first quartile and third quartile).

The inferential analysis of the association between the variables was performed using Fisher's Exact Test and Pearson's Chi-Square Test. The distribution analysis of the quantitative variables was performed using the Shapiro-Wilk test, and all variables had non-normal distribution. The inferential analysis of the quantitative variables as a function of nominal qualitative variables of two categories (independent groups) was performed using the Mann-Whitney test. The agreement analysis between the quantitative variables was performed using the Intraclass Correlation Coefficient test and between the qualitative variables using the Kappa test.

## RESULTS

This research was carried out between August 2018 and December 2019. The sample of the present study consisted of 382 pregnant women, divided into two groups: Not Exposed to Pesticides - 62 pregnant women not exposed to pesticides - and Exposed to Pesticides - 320 pregnant women exposed to pesticides. The groups and the number of participants in each stage of the validation study varied. The 10^th^ Health Region, which covers the west region of the State of Paraná, had greater participation (n=320; 84%) than the 4^th^ Health Region, corresponding to the southeast region of the state (n=62; 16%).

Considering only the Exposed to Pesticides Group, the average age of the participants was 26 years and six months, and the trimesters of pregnancy showed greater participation of pregnant women in the 3^rd^ trimester (n=126; 39.4%). The majority of the participants were Brazilian (n=310; 96.9%) and the most frequent level of education was complete high school (n=93; 29.1%). Only 17.8% of all the participants were studying at the time of the research. The family income of 31.6% of the participants was less than a salary (R$975.00), and between one and two salaries for 44.7%. Regarding previous pregnancies, an average of two pregnancies was observed. Most of the interviewees reported that they lived in an agricultural area (n=204; 63.75%).

Regarding the occupation of the women in the Exposed to Pesticides Group, 54.38% (n=174) reported working since they became pregnant, while 45.63% (n=146) deny having worked. Of those who indicated having worked, when asked if their work involves agriculture, 25.63% (n=82) answered yes and 28.75% (n=92) no; another 146 (45.63%) pregnant women did not answer this question, as a questionnaire criterion, since they indicated they were not working during pregnancy. When asked if they currently work, 60.94% (n=195) answered yes and 39.06% (n=125) no; 72.19% (n=231) reported that they were working a year ago, against 27.81% (n=89), who answered not being working in this period. Regarding when they stopped working, 6.25% (n=20) reported that they stopped working before knowing about the pregnancy, 32.19% (n=103) after knowing about the pregnancy, and another 197 (61.56%) answered that they did not have interrupted their work.

As for occupational planning, 102 (31.88%) reported that they would stop working only when the doctor determined or when they were no longer able to work, while 97 (30.31%) intended to work until they gave birth. 120 (37.50%) pregnant women still did not know when they would stop working. After giving birth, 251 (78.44%) intended to work, 16.56% (n=53) did not intend to work, and 5% (n=16) did not yet know. Regarding the return to work, 35.63% (n=114) believed returning between 3 months after giving birth, 9.38% (n=30) between 3 and 6 months after giving birth, 7.5% (n=24) 6 months after giving birth, and 47.5% (n=152) still did not know.

Concerning the medical resources used to perform prenatal care, more than 70% of the participants reported using the Unified Health System, followed by supplementary health (23%). In this question, the participants could choose more than one alternative if they used more than one service. As for the number of prenatal consultations, the average was 2.51 times. Regarding the month of the first visit to the doctor, it occurred at the 2.16 months of gestation.

The characterization of the scores for Knowledge, Attitudes and Practices was made from a determined score, based on the percentage of questions answered correctly, indicating that the higher the average, the greater the degree of knowledge, or attitudes and practices carried out. The analysis of Knowledge allowed verifying a significant level of knowledge of pregnant women about all related items, and most of the interviewees agreed that the damage caused by pesticides affects different populations, regardless of whether they are farmers or not, as well as recognized the main routes of poisoning and its symptoms.

Most of the pregnant women interviewed did not receive training on pesticides (n= 300; 93.75%) and only 20 pregnant women (6.25%), who received training, reported that it was offered by the companies that supply the products, companies/cooperatives linked to their job, and syndicates, about 2 years ago. Only these women answered questions about the topics discussed in the training.

The analysis of Attitudes showed a low mean score for the items “Use of appropriate clothing at work”, “Use of pesticides and care”, “Reasons for using pesticides at home” and “Reasons for using pesticides at work”. And regarding “Attitudes taken at home”, such as care with washing fruits and vegetables before eating, “Harmful attitudes for the fetus”, “Responsibility for the safe use of pesticides, reading the packaging label”, as well as “Responsibility for safe use of pesticides, reuse of packaging”, this was characterized as a safe attitude that demonstrates responsibility regarding the use of pesticides by most pregnant women.

The analysis of Practices for the safe use of pesticides refers to the use of protective equipment, which includes precautionary practices at home and at work, as effective ways to prevent exposure to pesticides, revealed safe practices in the three trimesters of pregnancy.

The results for validity, which refer to the fact that the instrument measures exactly what it proposes to measure, were obtained from the analysis of the three main types of validity: content validity, criterion validity, and construct validity.

### Content validity

To analyze content validity, the Content Validity Index^(11)^ was used. Three judges analyzed each question assigning a score from 1 to 4, where: 1 = unclear item; 2 = slightly unclear item; 3 = quite clear item; and 4 = very clear item^(9)^. The count of the number of questions with grades three and four was done and the total count was divided by the total number of questions evaluated. The calculation was performed for each judge individually and for the total number of judges. The results showed that the Content Validity Index was between 0.94 and 0.97 for the individual judges and 0.96 for the total of judges, which demonstrated acceptable agreement among the members of the committee of experts^(11)^.

### Criterion validity

For the criterion validity analysis, the domain scores of each construct were compared between two independent groups, constituted from the answers to questions that analyze the same construct. For this, participants were instructed to answer yes or no to the questions: a) “Do you believe you have adequate knowledge about the effects of pesticides?”; b) “Do you believe you have adequate attitudes towards pesticides?”, and c) “Do you believe you have safe practices regarding pesticides?”. Due to the non-normal distribution of the scores, the non-parametric Mann-Whitney test was used to compare the two independent groups. In addition, for the nominal qualitative variables of domains or questions of the construct Knowledge, an association between the categories of answers to the questions and the categories of answers to the domains or questions of the construct (Tables 1, 2, 3) was made. It is observed, in this analysis, that there was no difference or association between the criterion questions and the domains or questions of the KAP questionnaire.

**Table 1:** Comparison of the scores of the questions of the Knowledge construct of the KAP Questionnaire according to the independent groups, constituted from the answers to a question that evaluates the same construct in female farmers from the Exposed to Pesticides Group

Variable	Do you believe you have adequate knowledge about the effects of pesticides?	Mean	SD	Q25	Median	Q75	p-value
Knowledge about the damage of pesticides to human health	Yes	60.19	24.42	50.00	50.00	50.00	0.577
	No	58.33	23.07	50.00	50.00	50.00	
Knowledge about at risk population	Yes	89.51	23.74	83.33	100.00	100.00	0.524
	No	91.27	22.18	100.00	100.00	100.00	
Knowledge about poisoning pathways	Yes	70.56	23.44	60.00	80.00	80.00	0.054
	No	72.38	30.72	60.00	80.00	100.00	
Knowledge of intoxication symptoms	Yes	75.10	27.30	55.56	83.33	100.00	0.734
	No	76.98	24.92	66.67	77.78	100.00	
Knowledge about the risks of pesticides	Yes	58.91	21.31	43.75	56.25	81.25	0.160
	No	62.20	25.99	43.75	68.75	81.25	
Knowledge about the impact on health	Yes	60.49	26.63	50.00	66.67	83.33	0.964
	No	60.32	27.85	50.00	66.67	83.33	

Mann-Whitney test

**Caption:** SD = Standard deviation; Q25 = First quartile; Q75 = Third quartile

**Table 2:** Comparison of the scores of the questions of the Attitudes construct of the KAP Questionnaire according to the independent groups constituted from the answers to a question that evaluates the same construct in female farmers from the Exposed to Pesticides Group

Variable	Do you believe you have adequate attitudes towards pesticides?	Mean	SD	Q25	Median	Q75	p-value
Attitudes at home	Yes	64.08	12.83	60.00	60.00	60.00	0.567
	No	62.77	11.21	60.00	60.00	60.00	
Wearing appropriate clothing at work	Yes	29.52	30.08	0.00	20.00	60.00	0.176
	No	14.17	17.17	0.00	20.00	20.00	
Harmful attitudes to the fetus	Yes	81.12	23.42	75.00	87.50	100.00	0.849
	No	82.98	19.82	84.38	87.50	100.00	
Responsibility for the safe use of pesticides, read the packaging label	Yes	94.90	22.12	100.00	100.00	100.00	0.503
	No	92.55	26.39	100.00	100.00	100.00	
Responsibility for the safe use of pesticides, reuse of packaging	Yes	72.45	44.91	0.00	100.00	100.00	0.616
	No	69.15	46.44	0.00	100.00	100.00	
Use of pesticides and care	Yes	36.73	13.75	28.57	42.86	42.86	0.591
	No	38.26	13.11	28.57	42.86	42.86	
Reasons for using pesticides at home	Yes	32.16	22.58	11.11	33.33	44.44	0.670
	No	32.79	19.50	11.11	33.33	44.44	
Reasons for using pesticides at work	Yes	14.29	15.73	0.00	16.67	33.33	0.489
	No	16.29	15.03	0.00	16.67	29.17	

Mann-Whitney test

**Caption:** SD = Standard deviation; Q25 = First quartile; Q75 = Third quartile

**Table 3:** Analysis of the scores of the questions of the Practices construct of the KAP Questionnaire according to the independent groups constituted from the answers to a question that evaluates the same construct in female farmers of the Exposed to Pesticides Group

Variable	Do you believe you have safe practices regarding pesticides?	Mean	SD	Q25	Median	Q75	p-value
Precautionary practices	Yes	90.28	11.95	85.71	85.71	100.00	0.608
	No	90.36	13.50	85.71	100.00	100.00	
Safe practices at home	Yes	76.17	24.19	60.00	80.00	100.00	0.578
	No	78.81	22.46	60.00	80.00	100.00	
Practices related to domestic animals	Yes	39.58	20.45	50.00	50.00	50.00	0.181
	No	43.75	20.04	50.00	50.00	50.00	

Mann-Whitney test

**Caption:** SD = Standard deviation; Q25 = First quartile; Q75 = Third quartile

### Construct validity

For the construct validity analysis, the pregnant women in the Exposed to Pesticides Group were compared to the pregnant women in the Not Exposed to Pesticides Group, in order to verify differences in the scores of the domains of the constructs of Knowledge, Attitudes and Practices of the KAP questionnaire. The analysis allowed inferring that the groups were homogeneous for the variables age, nationality, education, and family income (Table 4).

**Table 4:** Descriptive analysis of the quantitative characterization variables in pregnant women in the Exposed and Not Exposed to Pesticides Group

Variable	Group			n		Mean		SD	Q25	Median	Q75	p-value
Age	Pregnant Woman Exposed			320		26.59		6.22	22.30	26.31	32.21	0.445
	Pregnant Woman not Exposed			62		25.89		6.90	21.14	24.55	31.98	
Nationality	Brazilian	n	310		62		0.377					
		%	96.9%		100.0%							
	Foreign	n	10		0							
		%	3.1%		0.0%							
Level of Education	Early childhood education (1-4 years)	n	16		4		<0.001*					
		%	5.0%		6.5%							
	Incomplete Early childhood education (1-4 years)	n	8		0							
		%	2.5%		0.0%							
	Primary education (5-8 years)	n	38		6							
		%	11.9%		9.7%							
	Incomplete Primary education (5-8 years)	n	58		8							
		%	18.1%		12.9%							
	Secondary education	n	93		11							
		%	29.1%		17.7%							
	Incomplete Secondary education	n	28		10							
		%	8.8%		16.1%							
	Technical education	n	1		10							
		%	0.3%		16.1%							
	Higher Education	n	58		4							
		%	18.1%		6.5%							
	Incomplete Higher Education	n	20		9							
		%	6.3%		14.5%							
Family income	<1 salary	n	101		27		0.081					
		%	31.6%		43.5%							
	1-2 salaries	n	143		24							
		%	44.7%		38.7%							
	2-3 salaries	n	21		0							
		%	6.6%		0.0%							
	SC	n	55		11							
		%	17.2%		17.7%							

Mann-Whitney test

**Caption:** n = Relative frequency; SD = Standard deviation; Q25 = First quartile; Q75 = Third quartile; * Significant p≤0.01

In the analysis of the scores of the questions of the constructs Knowledge, Practices and Attitudes of the KAP questionnaire, according to the groups of female farmers, it was observed that there was a statistical difference between the groups, and only for the domain “Attitudes in home” (p<0.001) the Exposed to Pesticides Group had a lower score than the Not Exposed to Pesticides Group (Table 5).

**Table 5:** Analysis of the scores of the questions of the constructs “Knowledge, Attitudes and Practices” according to the groups of female farmers

Variable	Group	n	Mean	SD	Q25	Median	Q75	p-value
Knowledge about the damage of pesticides to human health	Pregnant Women (Exposed)	320	58.91	24.22	50.00	50.00	50.00	0.060
	Pregnant Women (Not Exposed)	62	64.52	34.34	50.00	50.00	100.00	
Knowledge about at risk population	Pregnant Women (Exposed)	320	89.84	23.79	83.33	100.00	100.00	<0.001*
	Pregnant Women (Not Exposed)	62	81.18	23.66	66.67	91.67	100.00	
Knowledge about poisoning pathways	Pregnant Women (Exposed)	320	71.81	27.05	60.00	80.00	80.00	0.188
	Pregnant Women (Not Exposed)	62	72.26	32.86	60.00	80.00	100.00	
Knowledge of intoxication symptoms	Pregnant Women (Exposed)	320	74.69	26.12	66.67	77.78	100.00	<0.001*
	Pregnant Women (Not Exposed)	62	54.84	37.74	22.22	44.44	100.00	
Knowledge about the risks of pesticides	Pregnant Women (Exposed)	320	59.65	22.90	43.75	68.75	81.25	0.564
	Pregnant Women (Not Exposed)	62	57.16	25.43	37.50	62.50	75.00	
Knowledge about the impact on health	Pregnant Women (Exposed)	320	60.42	26.44	50.00	66.67	83.33	0.405
	Pregnant Women (Not Exposed)	62	58.06	25.74	50.00	66.67	66.67	
Attitudes at home	Pregnant Women (Exposed)	320	63.63	12.54	60.00	60.00	60.00	<0.001*
	Pregnant Women (Not Exposed)	62	89.68	16.89	80.00	100.00	100.00	
Wearing appropriate clothing at work	Pregnant Women (Exposed)	91	24.40	24.18	0.00	20.00	40.00	-
	Pregnant Women (Not Exposed)	0						
Harmful attitudes to the fetus	Pregnant Women (Exposed)	320	82.46	21.45	87.50	87.50	100.00	<0.001*
	Pregnant Women (Not Exposed)	62	69.96	19.40	62.50	75.00	87.50	
Responsibility for the safe use of pesticides, read the packaging label	Pregnant Women (Exposed)	320	94.06	23.67	100.00	100.00	100.00	<0.001*
	Pregnant Women (Not Exposed)	62	40.32	49.45	0.00	0.00	100.00	
Responsibility for the safe use of pesticides,reuse of packaging	Pregnant Women (Exposed)	320	74.06	43.90	0.00	100.00	100.00	0.041
	Pregnant Women (Not Exposed)	62	61.29	49.11	0.00	100.00	100.00	
Use of pesticides and care	Pregnant Women (Exposed)	209	38.82	13.38	28.57	42.86	42.86	<0.001*
	Pregnant Women (Not Exposed)	10	2.86	904	0.00	0.00	0.00	
Reasons for using pesticides at home	Pregnant Women (Exposed)	201	31.23	20.59	11.11	33.33	44.44	-
	Pregnant Women (Not Exposed)	0						
Reasons for using pesticides at work	Pregnant Women (Exposed)	148	15.54	14.76	0.00	16.67	29.17	-
	Pregnant Women (Not Exposed)	0						
Precautionary practices	Pregnant Women (Exposed)	320	89.38	14.60	85.71	85.71	100.00	<0.001*
	Pregnant Women (Not Exposed)	62	75.81	28.17	71.43	85.71	85.71	
Safe practices at home	Pregnant Women (Exposed)	224	78.30	22.31	60.00	80.00	100.00	-
	Pregnant Women (Not Exposed)	0						
Practices related to domestic animals	Pregnant Women (Exposed)	320	42.50	20.34	50.00	50.00	50.00	0.003*
	Pregnant Women (Not Exposed)	62	50.00	0.00	50.00	50.00	50.00	

Mann-Whitney test

**Caption:** n = Relative frequency; SD = Standard deviation; Q25 = First quartile; Q75 = Third quartile; * Significant p≤0.01

Fisher's Exact Test and Pearson's Chi-Square Test were used to associate the domains or nominal qualitative questions and the groups. It was observed that there was an association between the “Knowledge about other effects of pesticides” domain and the answer no in the Exposed to Pesticides Group (p=0.012).

## DISCUSSION

The KAP questionnaire is used worldwide to assess Knowledge, Attitudes and Practices on various subjects and in different populations. The questionnaire differs according to the area and type of study, having already been applied to pregnant women to investigate prenatal care and routine exams^(12,13)^.

Although there are few studies, mainly in Brazil, using protocols similar to KAP, some inferences can be made from the results obtained from the validation process carried out in the present study. When we compare the data from this research to the values obtained in other protocols, with other populations, we observe some controversies regarding both knowledge, attitudes and practices, as well as the validation process, which will be discussed throughout this discussion^(6,7)^.

In the demographic analysis of the group of pregnant women exposed to pesticides, it was noted that the average age was 26 years and six months, with greater participation of pregnant women in the 3rd trimester (n=126; 39.4%); the most frequent level of education was complete high school (n=93; 29.1%), and only 17.8% of the total number of participants was studying at the time of the research.

The family income of approximately 32% of the participants was less than one minimum wage (R$975.00), the current value at the time of the research, and between one and two minimum wages for 44.7%. According to the last census carried out in 2014, the State of Paraná has about 17% of its population in rural areas, and gains economic prominence with the 5^th^ highest HDI and the 6^th^ highest average real salary per inhabitant of the federative units, with an average per capita income of R$ 2,552.00. This data is in accordance with the analysis of the country's economic growth, which, taking GDP, per capita income and the sectoral production of the federative entities as a reference, the South and Southeast regions account for more than 70% of production and income, having 56% of the Brazilian population^(14)^.

In the pregnant women who participated in this study, a low average family income was noted, which negatively interferes with their access to health services, since family income and maternal education are considered the main determinants for adequate prenatal care^(15)^. Evidence indicates that, although almost all Brazilian pregnant women (98%) begin prenatal care, the better the women's income, the greater their participation in prenatal procedures and exams^(16)^.

The findings for the Knowledge items were similar to the results found in the previously developed study about the translation of the questionnaire, in which pregnant women, in general, demonstrated satisfactory knowledge about pesticides, and the longer the gestational trimester, the greater the knowledge^(6,7)^. The use of the KAP questionnaire also proved to be efficient in the assessment of educational measures in pregnant women regarding other subjects, such as gestational care and smoking^(17,18)^. Most of the interviewees agreed that the damage caused by pesticides affects different populations, regardless of whether they are farmers or not, as well as recognized the main routes and signs and symptoms of intoxication.

In the items related to knowledge about the damage caused by pesticides to human health and the risks of pesticides, the average was lower, however, it remained above 50%. The pregnant women in this study also did not demonstrate knowledge (63.75%) or did not report knowledge (23.75%) about other effects that pesticides can cause, in addition to those suggested by the questionnaire.

Pesticides affect human health directly and indirectly, as well as the environment in general, causing an imbalance in biomes. However, the totality of its impacts is not clearly defined and known yet, due to the multiplicity of factors involved. In this sense, knowledge about contamination risks is closely related to the way in which these populations relate to existing dangers, processes that are strongly biased by determinants of social, cultural, and economic orders^(17)^.

According to the literature, signs and symptoms of intoxication differ between acute and chronic, classified as mild, moderate, and severe. Among the symptoms of acute intoxication are headache, irritation, irritant or hypersensitivity contact dermatitis, nausea, vomiting, abdominal cramps, dizziness, generalized weakness, increased salivation and sweating, hypotension, cardiac arrhythmias, respiratory failure, acute pulmonary edema, seizures, changes in consciousness, shock, and coma, which may progress to death. Chronic symptoms manifest themselves through numerous pathologies that affect various organs and systems, with emphasis on immunological, hematological, hepatic, and neurological problems, congenital malformations, and tumors^(19)^.

For the recognition of risks and, therefore, the development of appropriate care, it is fundamental to carry out activities and/or educational programs that guide, clarify and teach strategies and safe forms of care. In the sample of this study, most pregnant women interviewed did not receive training on pesticides (93.75%), and only 20 pregnant women (6.25%), who received it, reported that it was offered through the companies that supply the products, companies/cooperatives linked to their job, and syndicates, about 2 years ago. Only the interviewees who reported having received training answered the questions about these topics.

These data conflict with the findings on the knowledge of pregnant women about pesticides, since they had high levels of knowledge, however, they did not receive training on the products. This may indicate that possibly this knowledge does not come from instruction or training, but something acquired through other means, such as information on television, newspapers, and the internet, or acquired through popular and intergenerational knowledge, since many of them come from farming families.

In recent years, with the proposal of the Family Health Strategy Program, which brings the general population closer to health care units, prenatal care coverage has been intensified, reducing risk pregnancies and childbirth complications, in addition to campaigns in the pre-and neonatal periods^(20)^. Specifically, in the state of Paraná, several government initiatives have been carried out to better assist this population, such as the program called “Mãe Paranaense”, which proposes the organization of maternal and child care in prenatal and puerperal actions and follow-up of the child growth and development, especially in the first year of life^(21)^.

In this sense, it is assumed that pregnant women who undergo prenatal care and participate in guidance, lectures, and conversations with health professionals, have more knowledge about gestational care and are alert to possible risks and take precautions for the comprehensive care for their fetus, even though pesticide is not the target topic.

Advances in the health area and evidence-based practice give rise to the constant need for valid and reliable measures, using calibrated instruments, to measure reality according to standards. In Brazil, the number of cross-cultural adaptations of instruments designed and validated in other cultures and the number of constructions of new questionnaires has increased significantly, supported by international educational institutions and funding from government agencies, since a large part of this research is aimed at improving the health condition of the general population^(22)^.

Assessment instruments are part of clinical practice and research in different areas of knowledge, and the assessment of their quality is essential for selecting instruments that provide valid and reliable measurements for their target population, respecting their particularities^(23)^.

Content validity refers to the judgment on the instrument, carried out by different expert examiners, who analyze the representativeness of the items in relation to the content areas and the relevance of the objectives to be measured^(10)^. In this study, the selected judges, who were invited to participate spontaneously in this study, were invited, as they develop research related to the theme of this study.

To analyze content validity, the Content Validity Index^(11)^ was used. Three judges analyzed each question assigning a score from 1 to 4, where: 1 = unclear item; 2 = slightly unclear item; 3 = quite clear item; and 4 = very clear item^(9)^. The count of the number of questions with grades three and four was done and the total count was divided by the total number of questions evaluated. The calculation was performed for each judge individually and for the total number of judges. The results showed that the Content Validity Index was between 0.94 and 0.97 for the individual judges and 0.96 for the total of judges, which demonstrated acceptable agreement among the members of the committee of experts, indicating that the evaluated items of the questionnaire have valid and accurate measures^(11)^.

Criterion validity is the existing correlation between the measure evaluated in relation to another measure or instrument that serves as an evaluation criterion, which has the same or similar attributes, and consists of the relationship between scores of a given instrument and some external criterion^(10)^.

In the case of this study, for the analysis of criterion validity, the domain scores of each construct were compared between two independent groups constituted from the answers to questions that analyze the same construct. After applying the questionnaire, three questions were tested in order to confirm the criterion validity^(10)^. For this, participants were instructed to answer yes or no to the questions: a) “Do you believe you have adequate knowledge about the effects of pesticides?”; b) “Do you believe you have adequate attitudes towards pesticides?”, and c) “Do you believe you have safe practices regarding pesticides?”.

The results showed that there was no difference or association between the criterion questions and the domains or questions in the questionnaire. In the case of the KAP questionnaire, no gold standard questionnaire was found for comparison. The original questionnaire in English is not validated and, for this reason, it does not present the analyzes for such a comparison.

It is possible that the established criterion, by presenting direct questions with objective answers, did not favor, being a weak criterion of comparison, since it limits the answers of the pregnant women. An alternative to be considered is to create a scale that allows more flexibility in responses. Furthermore, it is also believed that this was a reflection, after having answered all the questions in the questionnaire and having rethought about their knowledge, attitudes and practices.

In the literature, similar situations can be seen, as was the case with the validation of the “Lingual Frenulum Protocol for Infants”, which in the comparison of criterion validity, as no fully validated protocols, considered the gold standard in the literature, were found, the criterion validity analysis was performed by means of comparison with another protocol, which evaluated similar questions^(24)^. The variation in the strength of the correlation from medium to strong, found for the aspects considered in this research, was explained by the differences between the items and the way of evaluating the two protocols (Tables 1, 2, 3)^(25)^. These studies indicate, in general, that there are other possibilities to determine the validity criteria, indicating new possibilities to test the validity of this questionnaire.

To verify construct validity, several tests are carried out, which need to be analyzed in all their details. This type of validation aims to detect, among other aspects, which variables the test scores correlate with, which types of items make up the test, the degree of stability of the scores under varied conditions and the degree of homogeneity (whether the test measures a single trait or if, on the contrary, it measures several traits) of the test, with a view to having elements that can clarify the meaning of the instrument^(10)^.

In the case of this study, construct validity is fundamental, since it helps the researcher to determine and better understand the cognitive and psychological issues that are being measured by the test^(10)^. That is, we sought to verify the knowledge, attitudes and practices of pregnant women exposed to pesticides regarding these products. Construct validity is subdivided into three types: hypothesis testing, structural or factorial validity, and cross-cultural validity^(9-11)^.

In the hypothesis test, one of the strategies of this testing is the technique of known groups, in which different groups of individuals fill out the research instrument and, then, the results of the groups are compared^(11)^. In this study, for this analysis, the pregnant women in the Exposed to Pesticides Group were compared to the pregnant women in the Not Exposed to Pesticides Group to verify differences in the scores of the domains of the constructs of knowledge, attitudes and practices of the KAP Questionnaire. The analysis allowed inferring that the groups were homogeneous for the variables age, nationality, study, and family income. The analysis of the scores of the questions of the constructs Knowledge, Practices and Attitudes of the KAP Questionnaire showed that there was a statistical difference between the groups of pregnant women and only in the domain “Attitudes at home” the Exposed to Pesticides Group presented a lower score than the Not Exposed to Pesticides Group. This identifies that the pregnant women in the Not Exposed to Pesticides Group are more careful with cleaning fruits and vegetables before eating, with their work clothes, and with their own homes. Regarding this, when analyzing the questions regarding family income and education level, which could be factors that interfere in this question, it is observed that, in this study, these variables are homogeneous.

However, the Exposed to Pesticides Group presented higher scores than the Not Exposed to Pesticides Group in the domains “knowledge about the population at risk”, “knowledge of intoxication symptoms”, “harmful attitudes towards the fetus”, “responsibility for the safe use of pesticides, read the packaging label”, “responsibility for the safe use of pesticides, reuse of packaging”, “use of pesticides and care”, “precautionary practices”, “practices regarding domestic animals”. It is possible that, due to the fact that pesticides are not a reality close to unexposed pregnant women, they have no concern or knowledge about the subject and, perhaps, these subjects have never been discussed in the environment in which they live. With regard to exposed pregnant women, it is the opposite. Although the vast majority of them have not been instructed on the safe use and care of products, this topic is part of their experience and, indirectly, is being discussed by product sellers, agronomists, syndicate representatives and by their own families, since there is a common and general sense that “poison is harmful”.

Following the steps of construct validation, structural or factorial validity provides tools to assess the correlations in a large number of variables, defining the factors, that is, the variables strongly related to each other11. In this study, the characteristics of the instrument did not allow performing the factor analysis, as the KAP Questionnaire presents a variation in its question models: it has questions with “yes” and “no” alternatives, with Likert and essay scales. Another peculiarity of the questionnaire is that the answer to a question, whether yes or no, directly interferes with whether you will answer the next question or section of the questionnaire. These singularities make the factor analysis difficult since it is not possible to categorize the data.

This step was carried out in a previous study^(7)^, which aimed to translate, adapt and develop the preliminary normative study of the KAP Questionnaire into Brazilian Portuguese. In that study, the KAP Questionnaire was translated into Brazilian Portuguese, being analyzed by judges from areas related to the object of the study, allowing the revision and adequacy of the terms. Afterwards, the questionnaire was applied in a pilot group, in order to carry out the semantic analysis and cross-cultural adaptation of the terms. After adaptation, the instrument was back-translated into English. From the results, it was possible to verify that this instrument was coherent and satisfactory for surveying the knowledge, attitudes and practices of Brazilian pregnant women in relation to pesticides. In the present study, there were significant differences between knowledge and the stage of pregnancy, and the longer the pregnancy, the greater the knowledge about the risks of exposure to pesticides, as well as the adoption of safe attitudes and practices during the gestational period, at home and at work.

Concluding this discussion, it should be noted that, in this study, the KAP Questionnaire was validated in pregnant women exposed to pesticides, through content, criterion, and construct validity. The research, developed in two health regions in the State of Paraná, did not effectively count on all exposed pregnant women, which would express the cultural reality of the regions. Another limitation concerns the characteristics of the questionnaire, which, as it does not follow a pattern, did not allow for important analyses, such as exploratory factor analysis.

On the other hand, the significant number of pregnant women who participated in the study, and the fulfillment of all reliability and validity stages, allowed obtaining reliable data and the inference that the instrument is valid for application in Brazilian populations. Another bias to be highlighted is that despite the favorable validity results, indicating the possibility of using this questionnaire to survey the Knowledge, Attitudes and Practices of pregnant women farmers in Brazil, its application in the form of an interview, with the presence of the community health agents or another health professional, may have caused some embarrassment, which may have influenced the responses, especially regarding attitudes and practices taken at home.

Although the evidence that unsafe practices related to pesticides are associated with increased exposure of the Brazilian population to these products is not confirmed, it is possible that this is the main reason for the occurrence of the increase in changes generated by these risk behaviors, however, actual exposure measurements would be needed to confirm this hypothesis. This was not the focus of this study, but the responses obtained from the application of this instrument may be useful for future interventions.

Future research should aim to develop more homogeneous and reduced instruments, which facilitate the application in an even larger number of subjects, and present the complete analysis of its validation. Another focus of interest related to the theme would be the investigation of the men, partners of these pregnant women, and residents of the countryside, regarding their knowledge, attitudes and practices about the products, with regard to the concern with the pregnancy, since this issue does not should be a woman's sole concern.

The negative effects of pesticides on pregnant women and their newborns deserve attention from public health policies, since, as seen, research carried out in several countries proves that there are complications in pregnancy and to the health of the fetus. Scientific research has precisely the function of presenting specific situations so that, with effective public strategies, pesticides can stop causing problems to the population.

This study can be an initial path in understanding the reality of pregnant women and can be considered a guiding guide for future practices and actions aimed at the care of women exposed to pesticides. The validation of knowledge, attitude and practice assessment tools will help to develop useful and efficient programs.

## CONCLUSION

This study validated the KAP questionnaire considering its content, criterion, and construct. The developed analysis indicated that the psychometric properties of the cross-cultural adaptation of the Brazilian version of the scale are consistent and adequate for application in Brazil, which allows the recommendation to apply the instrument in a national context. It is important that the validated version of the questionnaire be applied in regions of Brazil to understand the cultural characteristics of each region, in order to propose effective measures for health promotion and damage prevention.
