# Supplementary material for: Validation of the Questionnaire “Knowledge, Attitudes and Practices” in populations of pregnant women exposed to pesticides
Source: Codas. 2023 Jun 26;35(3):e20210285. doi: 10.1590/2317-1782/20212021285en (PMC10348663; doi:10.1590/2317-1782/20212021285en)
Supplement: Translation

# Translation: Validação do Questionário “Conhecimento, Atitudes e Práticas” em populações de gestantes expostas a agrotóxicos

**DOI:** 10.1590/2317-1782/20212021285pt

**Authors:** Karlla Cassol, Cristiana Magni, Vanessa Veis Ribeiro, Maria Fernanda Capoani Garcia Mondelli, Andrea Cintra Lopes

**Affiliations:** 1 Programa de Pós-graduação em Fonoaudiologia, Faculdade de Odontologia de Bauru - FOB, Universidade de São Paulo - USP - Bauru (SP), Brasil.; 2 Universidade Estadual do Centro-Oeste - UNICENTRO - Irati (PR), Brasil.; 3 Universidade de Brasília - UnB - Brasília (DF), Brasil.

**Keywords:** Agricultura, Agroquímicos, Gestantes, Estudo de Validação, Protocolos Clínicos

## Abstract

**Objetivo:**

validar o questionário CAP em populações de gestantes expostas aos agrotóxicos no Estado do Paraná.

**Método:**

participaram do estudo 382 gestantes, divididas em dois grupos: Expostas a Agrotóxicos (n=320) e Não Expostas (n=62). O processo de validação envolveu a validade de conteúdo, critério e constructo. As etapas da pesquisa foram desenvolvidas entre agosto/2018 a dezembro/2019 na região oeste e centro-oeste do Paraná.

**Resultados:**

o instrumento demonstrou concordância aceitável na Validade de conteúdo por meio da avaliação de juízes; a Validade de critério por meio do critério estabelecido não apresentou associação; na análise da Validade de construto pela técnica de grupos conhecidos, demonstrou homogeneidade nas variáveis idade, nacionalidade e renda familiar.

**Conclusão:**

a análise desenvolvida indicou que as propriedades psicométricas da validação da versão brasileira da escala são consistentes e adequadas, o que permite a recomendação da aplicação do instrumento em contexto nacional.

## INTRODUÇÃO

A produção agrícola brasileira tem se tornado cada vez mais eficiente ao longo dos anos. Decorrente desse motivo, o setor econômico tem investido na fabricação e na comercialização de fertilizantes e agrotóxicos para o controle de pragas e ervas daninhas. Tal realidade faz com que se intensifiquem as discussões sobre os impactos da utilização indiscriminada de agrotóxicos e suas consequências na saúde pública^(1)^.

Dados da Agência Nacional de Vigilância Sanitária (ANVISA) colocam o Brasil como maior consumidor de agrotóxico mundial nas últimas safras, com perspectiva de aumentar a cada ano o uso intensivo de agrotóxicos nas lavouras^(2)^. Nesse ranking, o Paraná é o terceiro maior consumidor do Brasil, o que o torna um referencial válido para pesquisas relacionadas ao tema^(3)^.

Embora a discussão sobre os efeitos deletérios dos agrotóxicos no organismo das populações expostas direta ou indiretamente não seja recente, muito pouco se tem feito em relação à atenção e intervenção concreta sobre o seu uso. As ações que visam minimizar os impactos das intoxicações ainda são segregadas e pouco efetivas, dificultando o controle sobre o consumo e mau uso desses produtos químicos.

Nos últimos anos, os estudos se atêm a investigar os possíveis danos à saúde dos agricultores, com atenção principal às mulheres gestantes, caracterizadas como população de maior risco. As pesquisas nessa área apontam para complicações e intercorrências na gravidez, muitas vezes desconhecidas pela própria mulher. Essa questão transparece uma preocupação emergente de cuidado à saúde, mas principalmente evidencia o grande risco que uma população frágil, como a das gestantes, está exposta^(4)^.

Fato é que a existência de quaisquer intercorrências na gestação pode gerar desfechos indesejáveis com diferentes comprometimentos ao feto, como baixo peso ao nascimento, prematuridade e malformações congênitas, que são considerados fatores de risco para a mortalidade infantil^(4,5)^. Dentre esses fatores, que colocam em risco a saúde da gestante, incluem-se a exposição demasiada aos agrotóxicos, cujos malefícios já foram evidenciados por pesquisas experimentais, e seguem em andamento em seres humanos, permitindo antecipar a presença de alterações nos recém-nascidos, filhos de mães que estiveram expostas aos agrotóxicos antes e durante a gestação^(4,5)^.

Embora já existam comprovações sobre as consequências patológicas causadas pelos agrotóxicos, no sistema de saúde pública no Brasil são escassos protocolos que atendam, com eficiência e eficácia, as mulheres trabalhadoras expostas aos agrotóxicos durante o período pré-natal. Pode-se inferir que essa ausência se baseia na falsa ideia de que a mulher, por permanecer em casa, longe do trabalho agrícola, não estaria exposta aos efeitos danosos dos agrotóxicos. Indubitavelmente, a gestante que reside em áreas agrícolas ou próxima a elas, apresenta maior risco, quando comparada a outras gestantes, o que requer maior atenção e acompanhamento das políticas de saúde pública. Não somente são necessárias ações eficazes de cuidado, como também é urgente o estabelecimento de um protocolo que possa enquadrar esta gestante na categoria de alto risco, e não apenas no risco habitual, permitindo a ela o acesso a exames mais específicos que possam, inclusive, avaliar os níveis de toxicidade presentes no organismo.

A esse respeito, já existem em outros países, a preocupação quanto à exposição das gestantes aos agrotóxicos utilizados na agricultura, sendo que na Tailândia já estão sendo realizadas experiências com o Questionário Conhecimento, Atitudes e Práticas (CAP), por ser esse um instrumento eficaz para compreender os fatores associados com a exposição aos agrotóxicos no período pré-natal^(6)^.

Nesse sentido, foi realizada a tradução^(7)^ do Questionário CAP^(6)^, o qual se constitui como um instrumento que colabora para a identificação dos conhecimentos, atitudes e práticas das mulheres gestantes no que se refere à exposição aos agrotóxicos. Nesse estudo preliminar, realizado no Brasil, foi possível verificar associações significativas entre o conhecimento das mulheres e a fase da gestação, além de indicar comportamentos caracterizados como de riscos, bem como as práticas seguras diante dos produtos tóxicos. A partir dos resultados encontrados, também foi possível depreender que quanto mais tempo de gestação, maior é o conhecimento sobre os riscos da exposição aos agrotóxicos, como também a tomada de atitudes e práticas seguras durante o período gestacional, tanto em casa como no trabalho^(7)^.

Apesar dos resultados favoráveis à utilização do Questionário CAP para levantamento do conhecimento, das atitudes e das práticas de gestantes agricultoras no Brasil, para obter melhor confiabilidade é necessária a validação deste instrumento, a fim de verificar se o mesmo é válido para avaliar o que se propõe, utilizando uma amostra maior de participantes.

O objetivo deste estudo foi realizar a validação do Questionário CAP em populações de gestantes expostas a agrotóxicos no Estado do Paraná.

## MÉTODO

A presente pesquisa foi aprovada pelo Comitê de Ética em Pesquisa Envolvendo Seres Humanos do Centro Universitário Assis Gurgacz com coparticipação do Comitê de Ética em Pesquisa Envolvendo Seres Humanos da Secretaria de Estado da Saúde do Paraná (CEPSH/SESA/HT), sob parecer número 3.422.972. A amostra foi não probabilística por conveniência, em função da acessibilidade e disponibilidade da população. Foram recrutadas gestantes que frequentaram consultas e encontros pré-natais nas Unidades Básicas de Saúde de seus respectivos municípios, pertencentes a 4ª e a 10ª Regionais de Saúde, além de gestantes contatadas por meio de busca ativa, no período de Agosto de 2018 e Dezembro de 2019. Todos os indivíduos envolvidos (ou seus responsáveis) assinaram o Termo de Consentimento Livre e Esclarecido.

A amostra do estudo incluiu 382 gestantes de todos os períodos gestacionais, divididas em dois grupos: Grupo Não-Expostas a Agrotóxicos - 62 mulheres grávidas não-expostas a agrotóxicos; Grupo Expostas a Agrotóxicos - 320 mulheres grávidas expostas a agrotóxicos. As gestantes expostas são mulheres envolvidas direta ou indiretamente com a agricultura, sendo elas expostas aos agrotóxicos, alfabetizadas e com aparente condições emocionais e cognitivas de responder ao questionário sem auxílio. O Grupo Não-Exposta a Agrotóxicos foi composto por gestantes, sem vínculo direto ou indireto com a agricultura. Por se tratar da validação de um instrumento, necessitando de um grande número de sujeitos, a aplicação do instrumento foi realizada por profissionais da Atenção Básica em Saúde, mediante treinamento sobre os objetivos do instrumento e sua base teórica. O número de sujeitos em cada teste foi variável.

O questionário CAP aborda o Conhecimento, Atitudes e Práticas de gestantes agricultoras e inclui questões abrangentes sobre:

- O conhecimento sobre agrotóxicos - informação sobre o treinamento para uso dos produtos, rotas de exposição e riscos envolvidos, efeitos agudos e crônicos sobre a saúde, sintomas da toxicidade e métodos efetivos para prevenir a exposição.
- As atitudes sobre o uso dos agrotóxicos - informação sobre crenças para a responsabilidade de um uso seguro, susceptibilidade para efeitos sobre a saúde, efetividade dos agrotóxicos e razão para uso deles.
- As práticas para o uso seguro dos agrotóxicos - informação sobre o uso ocupacional e doméstico, uso de equipamento de proteção individual e uso de outras precauções seguras durante e depois da utilização dos agrotóxicos.

Para a validade^(8)^, que se refere ao fato de um instrumento medir exatamente o que se propõe a medir, há três aspectos que devem ser considerados: validade de conteúdo (o grau em que um teste ou instrumento de avaliação avalia todos os aspectos para os quais foi concebido); validade de critério (com que precisão um teste mede o resultado para o qual foi projetado para medir); e validade de construto^(9,10)^ (se as variáveis que estão sendo testadas se comportam de forma a sustentar a teoria).

Para a validade de conteúdo, foi selecionado um comitê de especialistas, composto por três juízes, pesquisadores da área de toxicologia, que estudam os efeitos dos agrotóxicos no organismo humano. Os mesmos avaliaram questão a questão utilizando a escala a Likert, atribuindo pontuação de 1 a 4, a cada item apresentado. Foram utilizadas opções mais curtas de análise, sendo elas: 1 = não claro, 2 = pouco claro, 3 = bastante claro, 4 = muito claro^(9)^. Após a análise, os itens que pontuaram 1 e 2 foram excluídos do instrumento, e os itens que pontuaram 3 e 4 foram somados e divididos pelo número total de resposta, gerando o índice de concordância entre os especialistas.

Referente a validade de critério, por não haver um instrumento padrão-ouro para análise de correlação, optou-se pela validade concorrente, com o uso de um critério estabelecido, sendo ele três perguntas breves, que avaliam o mesmo construto: “1- Você acredita que possui conhecimento adequado sobre os agrotóxicos?; 2 - Você acredita que possui atitudes adequadas frente aos agrotóxicos?; 3 - Você acredita que possui práticas seguras em relação aos agrotóxicos?”. As alternativas de respostas eram “sim” e “não”. Nessa etapa participaram 244 gestantes expostas a agrotóxicos^(10)^.

No que diz respeito a validade de construto, dada as características do questionário, não foi possível realizar a análise da validade convergente, pois o estudo base não apresenta testes de validação, nem a análise fatorial, pois as questões que compõem o questionário não seguem um padrão. Para elencar evidências necessárias, a fim de garantir a validação do construto, foi realizada a técnica de Grupos Conhecidos. Nessa técnica, o instrumento foi aplicado nos dois grupos: Grupo Expostas a Agrotóxicos, composto por 320 gestantes e Grupo Não Expostas a Agrotóxicos, composto por 62 gestantes^(9-11)^. Posteriormente, as respostas de ambos os grupos foram analisadas e comparadas a fim de verificar respostas diferentes entre eles.

Os dados foram analisados de forma descritiva e inferencial. Utilizou-se o software SPSS 25.0. A análise descritiva das variáveis qualitativas nominais foi realizada por frequência relativa e percentual. A análise descritiva das variáveis quantitativas discretas e contínuas, e qualitativas ordinais foi realizada por do cálculo de medidas de tendência central (média e mediana), variabilidade (desvio padrão) e posição (primeiro quartil e terceiro quartil).

A análise inferencial de associação entre as variáveis foi realizada com os Teste Exato de Fisher e Qui-Quadrado de Pearson. A análise de distribuição das variáveis quantitativas foi realizada com o teste Shapiro-Wilk, e todas obtiveram distribuição não-normal. A análise inferencial das variáveis quantitativas em função de variáveis qualitativas nominais de duas categorias (grupos independentes) foi realizada com o teste de Teste de Mann-Whitney. A análise da concordância entre as variáveis quantitativas foi realizada com o teste de Coeficiente de Correlação Intraclasse, e entre as variáveis qualitativas foi realizada com o teste de Kappa.

## RESULTADOS

A pesquisa foi realizada entre Agosto de 2018 e Dezembro de 2019. A amostra do presente estudo foi composta por 382 gestantes, divididas em dois grupos: Grupo Não Expostas a Agrotóxicos - 62 mulheres grávidas não expostas a agrotóxicos; Grupo Expostas a Agrotóxicos - 320 mulheres grávidas expostas a agrotóxicos. Os grupos e número de participantes em cada etapa do estudo de validação foi variável. A 10ª Regional de Saúde, que abrange a região Oeste do Estado do Paraná teve participação maior (n=320; 84%) que a 4ª Regional de Saúde, correspondente a região Sudeste do estado (n=62; 16%).

Considerando apenas o Grupo Expostas a Agrotóxicos, a idade média das participantes foi de 26 anos e seis meses, e os trimestres de gestação mostram uma maior participação de gestantes no 3° trimestre (n=126; 39,4%). A maioria das participantes era de nacionalidade brasileira (n=310; 96,9%) e o grau de escolaridade mais frequente foi o de ensino médio completo (n=93; 29,1%), sendo que apenas 17,8% do total das participantes encontravam-se estudando no momento da pesquisa. A renda familiar de 31,6% das participantes era de menos de um salário mínimo (R$ 975,00), valor vigente à época da pesquisa, e entre um e dois salários para 44,7%. Sobre as gestações anteriores, observou-se uma média de duas gestações. A maior parte das entrevistadas relatam que viviam em uma área agrícola (n=204; 63,75%).

Referente a ocupação das gestantes do Grupo Expostas a Agrotóxicos, 54,38% (n=174) relataram trabalhar desde que ficaram grávidas, enquanto que 45,63% (n=146) negam ter trabalhado. Das que assinalaram ter trabalhado, quando questionadas se o trabalho envolve a agricultura, 25,63% (n=82) responderam sim e 28,75% (n=92) não; outras 146 (45,63%) gestantes não responderam a essa questão, como um critério do questionário, uma vez que assinalaram não estar trabalhando na gravidez. Quando questionadas se atualmente trabalham, 60,94% (n=195) afirmaram, e 39,06% (n=125) negaram; 72,19% (n=231) relatam que há um ano atrás estavam trabalhando, contra 27,81% (n=89) que não. Relativo à quando parou de trabalhar, 6,25% (n=20) relatam que pararam antes de saber da gestação, 32,19% (n=103) após saber da gravidez, e outras 197 (61,56%) afirmam não ter interrompido o trabalho.

Sobre o planejamento ocupacional, 102 (31,88%) relataram que iriam parar de trabalhar apenas quando o médico determinasse ou quando não conseguissem mais trabalhar, enquanto que 97 (30,31%) pretendiam trabalhar até dar à luz, e outras 120 (37,50%) gestantes ainda não sabiam quando parariam. Após dar à luz, 251 (78,44%) das gestantes pretendiam trabalhar, 16,56% (n=53) não, e 5% (n=16) não sabiam ainda. Sobre o período de retorno ao trabalho, 35,63% (n=114) acreditavam retornar entre 3 meses após dar à luz, 9,38% (n=30) 3 e 6 meses após dar à luz, 7,5% (n=24) 6 meses após dar à luz, e 47,5% (n=152) ainda não tinham esse conhecimento.

Acerca dos recursos médicos utilizados para realização do pré-natal, o destaque foi o Sistema Único de Saúde, representado por mais de 70% das usuárias, seguido da saúde suplementar com 23%. Nessa questão, a gestante poderia assinalar mais de uma alternativa, caso utilizasse mais de um serviço. Referente a quantas consultas participou no pré-natal, a média foi de 2,51 vezes. Sobre o mês da primeira visita ao programa, este foi de 2,16 meses de gestação.

A caracterização dos escores para Conhecimento, Atitudes e Práticas foi feita a partir de um score determinado com base na porcentagem de questões respondidas corretamente, indicando que quanto maior a média, maior o grau de conhecimento, ou atitudes e práticas tomadas. A análise de Conhecimento permitiu verificar um nível de conhecimento significativo das gestantes sobre todos os itens relacionados, sendo que a maioria das entrevistadas concordaram sobre os danos dos agrotóxicos afetarem diferentes populações, independentemente de serem agricultores ou não, bem como, reconheceram as principais vias de intoxicação e seus sintomas.

A maioria das gestantes entrevistadas não receberam treinamento sobre os agrotóxicos (n= 300; 93,75%), e apenas 20 gestantes (6,25%) que o receberam, relatam que o mesmo foi realizado por intermédio das empresas fornecedoras dos produtos, empresas/cooperativas vinculadas ao seu emprego, e sindicatos, há cerca de 2 anos. Apenas essas mulheres responderam as questões sobre os tópicos discutidos no treinamento.

A análise sobre as Atitudes demonstrou média de acertos baixa nos itens “Uso de vestimenta adequada no trabalho”, “Uso de agrotóxicos e cuidados”, “Motivos para o uso de agrotóxicos em casa” e “Motivos para uso de agrotóxicos no trabalho”. E referente a “Atitudes tomadas em casa”, como cuidados com a lavagem de frutas e verduras antes de comer, “Atitudes prejudiciais para o feto”, “Responsabilidade para uso seguro dos agrotóxicos, ler rótulo das embalagens” bem como “Responsabilidade para uso seguro dos agrotóxicos, reutilização das embalagens” foi caracterizada como atitudes seguras e que demonstra responsabilidade quanto ao uso de agrotóxicos por grande parte das gestantes.

A análise sobre “Práticas” para o uso seguro dos agrotóxicos, que incluem práticas de precaução, em casa e no trabalho, como formas efetivas de prevenir a exposição a agrotóxicos, refere-se ao uso de equipamento de proteção e os resultados revelam práticas seguras nos três trimestres de gestação.

Os resultados para a Validade, que se referem ao fato do instrumento medir exatamente o que se propõe a medir, foi obtido a partir da análise dos três tipos principais de validade: validade de conteúdo, validade de critério e validade de constructo.

### Validade de conteúdo

Para analisar a validade de conteúdo, utilizou-se o Índice de Validade de Conteúdo^(11)^. Três juízes analisaram cada questão atribuindo uma pontuação de 1 a 4, em que: 1 = item não claro; 2 = item pouco claro; 3 = item bastante claro; e 4 = item muito claro^(9)^. Foi contabilizada a contagem do número de questões com notas três e quatro, e o total da contagem foi dividido pelo número total de questões avaliadas. O cálculo foi realizado para cada juiz individualmente, e para o total de juízes. Os resultados mostram que o IVC foi entre 0,94 e 0,97 para os juízes individuais, e de 0,96 para o total de juízes, o que demonstrou concordância aceitável entre os membros do comitê de especialistas^(11)^.

### Validade de critério

Para a análise da validade de critério, os escores dos domínios de cada construto foram comparados entre dois grupos independentes constituídos a partir das respostas para questões que analisam o mesmo construto. Para isso, os participantes foram orientados a responder “sim” ou “não” para as perguntas: a) “Você acredita que tem conhecimento adequado sobre os efeitos dos produtos agrotóxicos?”; b) “Você acredita que tem atitudes adequadas frente aos agrotóxicos?”, e c) “Você acredita que possui práticas seguras sobre os agrotóxicos?”. Devido a distribuição não-normal dos escores foi utilizado o teste não-paramétrico para comparação de dois grupos independentes Teste de Mann-Whitney. Além disso, para as variáveis qualitativas nominais de domínios ou perguntas do construto conhecimento, foi realizada associação entre as categorias de respostas às questões, e as categorias de resposta aos domínios ou perguntas do construto (Tabelas 1, 2, 3). Observa-se nessa análise que não houve diferença ou associação entre as questões critério, e os domínios ou perguntas do questionário CAP.

**Tabela 1:** Comparação dos escores das perguntas do construto “Conhecimento” do Questionário CAP em função dos grupos independentes constituídos a partir das respostas a uma pergunta que avalia o mesmo construto em agricultoras do Grupo Expostas

Variável	Você acredita que tem conhecimento adequado sobre os efeitos dos produtos agrotóxicos?	Média	DP	Q25	Mediana	Q75	p-valor
Conhecimento sobre o prejuízo dos agrotóxicos para a saúde humana	Sim	60,19	24,42	50,00	50,00	50,00	0,577
	Não	58,33	23,07	50,00	50,00	50,00	
Conhecimento sobre população de risco	Sim	89,51	23,74	83,33	100,00	100,00	0,524
	Não	91,27	22,18	100,00	100,00	100,00	
Conhecimento sobre vias de intoxicação	Sim	70,56	23,44	60,00	80,00	80,00	0,054
	Não	72,38	30,72	60,00	80,00	100,00	
Conhecimento dos sintomas de intoxicação	Sim	75,10	27,30	55,56	83,33	100,00	0,734
	Não	76,98	24,92	66,67	77,78	100,00	
Conhecimento sobre riscos dos agrotóxicos	Sim	58,91	21,31	43,75	56,25	81,25	0,160
	Não	62,20	25,99	43,75	68,75	81,25	
Conhecimento sobre o impacto à saúde	Sim	60,49	26,63	50,00	66,67	83,33	0,964
	Não	60,32	27,85	50,00	66,67	83,33	

Teste de Mann-Whitney

**Legenda:** DP = Desvio padrão; Q25 = Primeiro quartil; Q75 = Terceiro quartil

**Tabela 2:** Comparação dos escores das perguntas do construto “Atitudes” do Questionário CAP em função dos grupos independentes constituídos a partir das respostas a uma pergunta que avalia o mesmo construto em agricultoras do Grupo Expostas

Variável	Você acredita que tem atitudes adequadas frente aos agrotóxicos?	Média	DP	Q25	Mediana	Q75	p-valor
Atitudes em casa	Sim	64,08	12,83	60,00	60,00	60,00	0,567
	Não	62,77	11,21	60,00	60,00	60,00	
Uso de vestimenta adequada no trabalho	Sim	29,52	30,08	0,00	20,00	60,00	0,176
	Não	14,17	17,17	0,00	20,00	20,00	
Atitudes prejudiciais para o feto	Sim	81,12	23,42	75,00	87,50	100,00	0,849
	Não	82,98	19,82	84,38	87,50	100,00	
Responsabilidade para uso seguro dos agrotóxicos, ler rótulo das embalagens	Sim	94,90	22,12	100,00	100,00	100,00	0,503
	Não	92,55	26,39	100,00	100,00	100,00	
Responsabilidade para uso seguro dos agrotóxicos, reutilização das embalagens	Sim	72,45	44,91	0,00	100,00	100,00	0,616
	Não	69,15	46,44	0,00	100,00	100,00	
Uso de agrotóxicos e cuidados	Sim	36,73	13,75	28,57	42,86	42,86	0,591
	Não	38,26	13,11	28,57	42,86	42,86	
Motivos para uso de agrotóxicos em casa	Sim	32,16	22,58	11,11	33,33	44,44	0,670
	Não	32,79	19,50	11,11	33,33	44,44	
Motivos para uso de agrotóxicos no trabalho	Sim	14,29	15,73	0,00	16,67	33,33	0,489
	Não	16,29	15,03	0,00	16,67	29,17	

Teste de Mann-Whitney

**Legenda:** DP = Desvio padrão; Q25 = Primeiro quartil; Q75 = Terceiro quartil

**Tabela 3:** Análise dos escores das perguntas do construto “Práticas” do Questionário CAP em função dos grupos independentes constituídos a partir das respostas a uma pergunta que avalia o mesmo construto em agricultoras do Grupo Expostas

Variável	Você acredita que possui práticas seguras sobre os agrotóxicos?	Média	DP	Q25	Mediana	Q75	p-valor
Práticas de precaução	Sim	90,28	11,95	85,71	85,71	100,00	0,608
	Não	90,36	13,50	85,71	100,00	100,00	
Práticas seguras em casa	Sim	76,17	24,19	60,00	80,00	100,00	0,578
	Não	78,81	22,46	60,00	80,00	100,00	
Práticas referentes a animais domésticos	Sim	39,58	20,45	50,00	50,00	50,00	0,181
	Não	43,75	20,04	50,00	50,00	50,00	

Teste de Mann-Whitney

**Legenda:** DP = Desvio padrão; Q25 = Primeiro quartil; Q75 = Terceiro quartil

### Validade de constructo

Para a análise da validade de construto, as gestantes do Grupo Expostas a Agrotóxicos foram comparadas às gestantes do Grupo Não Expostas a Agrotóxicos, a fim de verificar diferenças nos escores dos domínios dos construtos de conhecimento, atitudes e práticas do Questionário CAP. A análise permite inferir que os grupos foram homogêneos para as variáveis idade, nacionalidade, estuda atualmente e renda familiar (Tabela 4).

**Tabela 4:** Análise descritiva das variáveis quantitativas de caracterização em gestantes do Grupo Expostas e do Grupo Não Expostas

Variável	Grupo			n		Média	DP	Q25	Mediana	Q75	p-valor
Idade	Gestante exposta			320		26,59	6,22	22,30	26,31	32,21	0,445
	Gestante não exposta			62		25,89	6,90	21,14	24,55	31,98	
Nacionalidade	Brasileira	n	310		62		0,377				
		%	96,9%		100,0%						
	Estrangeira	n	10		0						
		%	3,1%		0,0%						
Grau de escolaridade	Ensino Fundamental (1-4 anos)	n	16		4		<0,001*				
		%	5,0%		6,5%						
	Ensino Fundamental Incompleto (1-4 anos)	n	8		0						
		%	2,5%		0,0%						
	Ensino Fundamental (5-8 anos)	n	38		6						
		%	11,9%		9,7%						
	Ensino Fundamental Incompleto (5-8 anos)	n	58		8						
		%	18,1%		12,9%						
	Ensino Médio	n	93		11						
		%	29,1%		17,7%						
	Ensino Médio Incompleto	n	28		10						
		%	8,8%		16,1%						
	Curso Técnico	n	1		10						
		%	0,3%		16,1%						
	Ensino Superior	n	58		4						
		%	18,1%		6,5%						
	Ensino Superior Incompleto	n	20		9						
		%	6,3%		14,5%						
Renda familiar	<1 salário	n	101		27		0,081				
		%	31,6%		43,5%						
	1-2 salários	n	143		24						
		%	44,7%		38,7%						
	2-3 salários	n	21		0						
		%	6,6%		0,0%						
	SC	n	55		11						
		%	17,2%		17,7%						

Teste de Mann-Whitney

**Legenda:** n = Frequência relativa; DP = Desvio padrão; Q25 = Primeiro quartil; Q75 = Terceiro quartil; * Significante p≤0.01;

Na análise dos escores das perguntas dos construtos conhecimento, práticas e atitudes do Questionário CAP em função dos grupos em agricultoras, observou-se que houve diferença estatística entre os grupos, sendo que apenas para o domínio “Atitudes em casa” (p<0,001) o Grupo Expostas a Agrotóxicos apresentou menor escore que o Grupo Não Expostas a Agrotóxicos (Tabela 5).

**Tabela 5:** Análise dos escores das perguntas dos construtos “Conhecimento, atitudes e práticas” em função dos grupos em agricultoras

Variável	Grupo	n	Média	DP	Q25	Mediana	Q75	p-valor
Conhecimento sobre o prejuízo dos agrotóxicos para a saúde humana	Gestante exposta	320	58,91	24,22	50,00	50,00	50,00	0,060
	Gestante não exposta	62	64,52	34,34	50,00	50,00	100,00	
Conhecimento sobre população de risco	Gestante exposta	320	89,84	23,79	83,33	100,00	100,00	<0,001*
	Gestante não exposta	62	81,18	23,66	66,67	91,67	100,00	
Conhecimento sobre vias de intoxicação	Gestante exposta	320	71,81	27,05	60,00	80,00	80,00	0,188
	Gestante não exposta	62	72,26	32,86	60,00	80,00	100,00	
Conhecimento dos sintomas de intoxicação	Gestante exposta	320	74,69	26,12	66,67	77,78	100,00	<0,001*
	Gestante não exposta	62	54,84	37,74	22,22	44,44	100,00	
Conhecimento sobre riscos dos agrotóxicos	Gestante exposta	320	59,65	22,90	43,75	68,75	81,25	0,564
	Gestante não exposta	62	57,16	25,43	37,50	62,50	75,00	
Conhecimento sobre o impacto à saúde	Gestante exposta	320	60,42	26,44	50,00	66,67	83,33	0,405
	Gestante não exposta	62	58,06	25,74	50,00	66,67	66,67	
Atitudes em casa	Gestante exposta	320	63,63	12,54	60,00	60,00	60,00	<0,001*
	Gestante não exposta	62	89,68	16,89	80,00	100,00	100,00	
Uso de vestimenta adequada no trabalho	Gestante exposta	91	24,40	24,18	0,00	20,00	40,00	-
	Gestante não exposta	0						
Atitudes prejudiciais para o feto	Gestante exposta	320	82,46	21,45	87,50	87,50	100,00	<0,001*
	Gestante não exposta	62	69,96	19,40	62,50	75,00	87,50	
Responsabilidade para uso seguro dos agrotóxicos, ler rótulo das embalagens	Gestante exposta	320	94,06	23,67	100,00	100,00	100,00	<0,001*
	Gestante não exposta	62	40,32	49,45	0,00	0,00	100,00	
Responsabilidade para uso seguro dos agrotóxicos, reutilização das embalagens	Gestante exposta	320	74,06	43,90	0,00	100,00	100,00	0,041*
	Gestante não exposta	62	61,29	49,11	0,00	100,00	100,00	
Uso de agrotóxicos e cuidados	Gestante exposta	209	38,82	13,38	28,57	42,86	42,86	<0,001*
	Gestante não exposta	10	2,86	9,04	0,00	0,00	0,00	
Motivos para uso de agrotóxicos em casa	Gestante exposta	201	31,23	20,59	11,11	33,33	44,44	-
	Gestante não exposta	0						
Motivos para uso de agrotóxicos no trabalho	Gestante exposta	148	15,54	14,76	0,00	16,67	29,17	-
	Gestante não exposta	0						
Práticas de precaução	Gestante exposta	320	89,38	14,60	85,71	85,71	100,00	<0,001*
	Gestante não exposta	62	75,81	28,17	71,43	85,71	85,71	
Práticas seguras em casa	Gestante exposta	224	78,30	22,31	60,00	80,00	100,00	-
	Gestante não exposta	0						
Práticas referentes a animais domésticos	Gestante exposta	320	42,50	20,34	50,00	50,00	50,00	0,003*
	Gestante não exposta	62	50,00	0,00	50,00	50,00	50,00	

Teste de Mann-Whitney

**Legenda:** n = Frequência relativa; DP = Desvio padrão; Q25 = Primeiro quartil; Q75 = Terceiro quartil; * Significante p≤0.01.

Foi utilizado o Teste Exato de Fisher e o Teste Qui-Quadrado de Pearson para associar os domínios ou questões qualitativas nominais e os grupos. Observou-se que houve associação entre o domínio “conhecimento sobre outros efeitos dos agrotóxicos” e a resposta não no Grupo Expostas (p=0,012).

## DISCUSSÃO

O questionário CAP é usado mundialmente para avaliar o Conhecimento, as Atitudes e as Práticas de diversos assuntos e em diferentes populações. O instrumento se difere de acordo com a área e o tipo de estudo, já tendo sido aplicado em gestantes, a fim de investigar os cuidados no período pré-natal e exames de rotina^(12,13)^.

Embora existam poucas pesquisas, principalmente no Brasil, utilizando protocolos semelhantes a esse, algumas inferências podem ser feitas a partir dos resultados obtidos do processo de validação no presente estudo. Quando comparamos os dados dessa pesquisa aos valores obtidos em outros protocolos, com outras populações, observamos algumas controvérsias no que se referem tanto ao conhecimento, as atitudes e as práticas, quanto ao processo de validação, que serão comentadas no decorrer desta discussão^(6,7)^.

Na análise demográfica do grupo de gestantes expostas, notou-se que a média de idade foi de 26 anos e seis meses, com maior participação de gestantes no 3° trimestre (n=126; 39,4%); o grau de escolaridade mais frequente foi o de ensino médio completo (n=93; 29,1%), sendo que apenas 17,8% do total das participantes encontravam-se estudando no momento da pesquisa.

A renda familiar de aproximadamente 32% das participantes era de menos de um salário mínimo (R$ 975,00), valor vigente na época da pesquisa, entre um e dois salários para 44,7%. De acordo com o último censo realizado, em 2014, o Estado do Paraná possui cerca de 17% de sua população em área rural, e ganha destaque econômico com o 5º maior IDH e 6ª maior média salarial real habitual das unidades federativas, com uma renda média *per capita* de R$ 2.552,00. Esse dado vai de acordo com a análise de crescimento econômico do país, que, tomando como referência PIB, renda *per capita* e produção setorial dos entes federativos, as regiões Sul e Sudeste respondem por mais de 70% da produção e da renda, acomodando 56% da população brasileira^(14)^.

Nas gestantes que participaram desse estudo, notou-se renda média familiar baixa, e isso interfere negativamente no acesso dessas mulheres aos serviços de saúde, uma vez que renda familiar e escolaridade materna são considerados como os principais determinantes para a realização de pré-natal adequado^(15)^. Evidências apontam que apesar de quase a totalidade das gestantes brasileiras (98%) começar o pré-natal, quanto melhor a renda das mulheres, maior a participação nos procedimentos e exames relativos ao pré-natal^(16)^.

Os achados para os itens Conhecimento foram semelhantes ao estudo de tradução do instrumento, desenvolvidos anteriormente, onde as gestantes de maneira geral demonstraram conhecimento satisfatório sobre os agrotóxicos, sendo que quanto maior o trimestre de gestação, maior foi o conhecimento^(6,7)^. O uso do questionário CAP também demonstrou ser eficiente na avaliação de medidas educativas em gestantes, quanto a outros assuntos, como cuidados gestacionais e tabagismo^(17,18)^. A maioria das entrevistadas concordou que os danos dos agrotóxicos afetaram diferentes populações, independentemente de serem agricultores ou não, bem como, reconheceram as principais vias e sinais e sintomas de intoxicação.

Nos itens referentes ao conhecimento sobre o prejuízo dos agrotóxicos para a saúde humana e sobre riscos dos agrotóxicos a média apresentou-se mais baixa, entretanto, manteve-se acima dos 50%. As gestantes desse estudo também não demonstraram conhecer (63,75%) ou não relatam conhecimento (23,75%) sobre outros efeitos que os agrotóxicos podem causar, além dos sugeridos pelo questionário.

Os agrotóxicos afetam a saúde humana direta e indiretamente, bem como o meio ambiente em geral, ocasionando desequilíbrio nos biomas. Entretanto, nem todos os impactos são claramente definidos e conhecidos, devido a multiplicidade de fatores envolvidos. Nesse sentido, o conhecimento sobre os riscos de contaminação está intimamente relacionado à forma, através da qual estas populações se relacionam com os perigos existentes, processos estes fortemente enviesados por determinantes de ordens social, cultural e econômica^(17)^.

De acordo com a literatura, os sinais e sintomas de intoxicação diferem entre agudos e crônicos, classificados em leves, moderados e graves. Entre os sintomas de intoxicação agudos são caracterizados por cefaleia, irritação, dermatite de contato irritativa ou por hipersensibilidade, náusea, vômitos, cólicas abdominais, tonturas, fraqueza generalizada, salivação e sudorese aumentadas, hipotensão, arritmias cardíacas, insuficiência respiratória, edema agudo de pulmão, convulsões, alterações da consciência, choque, coma, podendo evoluir para óbito. Os sintomas crônicos manifestam-se por meio de inúmeras patologias que atingem vários órgãos e sistemas, com destaque para os problemas imunológicos, hematológicos, hepáticos, neurológicos, malformações congênitas e tumores^(19)^.

Para o reconhecimento dos riscos e, com isso, o desenvolvimento de cuidados apropriados, é fundamental a ocorrência de atividades e/ou programas educativos, que orientem, esclareçam e ensinem estratégias e formas seguras de cuidados. Na amostra deste estudo, a maioria das gestantes entrevistadas não recebeu treinamento sobre os agrotóxicos (93,75%), e apenas 20 gestantes (6,25%) que o receberam, relatam que o mesmo foi realizado por intermédio das empresas fornecedoras dos produtos, empresas/cooperativas vinculadas ao seu emprego, e sindicatos, há cerca de 2 anos. Apenas as entrevistadas que referiram ter tido treinamento é que responderam as questões sobre os tópicos discutidos nessa ocasião.

Esses dados conflitam com os achados sobre o conhecimento das gestantes sobre agrotóxicos, uma vez que elas apresentaram altos níveis de conhecimento, no entanto, não receberam treinamento sobre os produtos. Isso pode indicar, que possivelmente esse conhecimento não é advindo de uma instrução ou treinamento, mas algo adquirido por outros meios, como informativos em televisão, jornais e internet, ou adquiridos por meio do saber popular e intergeracional, já que muitas delas são oriundas de famílias agricultoras.

Nos últimos anos, com a proposta do Programa Estratégia de Saúde da Família, que aproxima a população, em geral, das unidades de cuidados à saúde, intensificou-se a cobertura de acompanhamento pré-natal, diminuindo as gestações de risco e complicações de parto, além das campanhas nos períodos pré e neonatal^(20)^. Especificamente, no estado do Paraná, diversas iniciativas governamentais têm sido executadas a fim de melhor assistir essa população, a citar o programa denominado “Mãe Paranaense”, que propõe a organização da atenção materno-infantil nas ações de pré-natal e puerpério, e acompanhamento do crescimento e desenvolvimento das crianças, em especial no primeiro ano de vida^(21)^.

Nesse sentido, supõe-se que gestantes que realizam o acompanhamento pré-natal e participam de orientações, palestras e conversas com os profissionais da saúde, apresentam mais conhecimento sobre os cuidados gestacionais e estão alertas com relação aos possíveis riscos e tomada de precauções para o cuidado integral com seu feto, mesmo o tema agrotóxicos não sendo o tema alvo.

O avanço na área da saúde e a prática baseada em evidências faz emergir a necessidade constante de medidas válidas e confiáveis, usando instrumentos calibrados, para medir construções aplicadas de acordo com os padrões. No Brasil, o número de adaptações de instrumentos elaborados e validados em outras culturas e o número de construções de novos questionários têm aumentado significativamente, apoiados por instituições educacionais internacionais e o financiamento de agências governamentais, uma vez que, grande parte dessas pesquisas, são destinadas a melhoria da condição de saúde da população geral^(22)^.

Os instrumentos de medida integram a prática clínica e a pesquisa em diferentes áreas do conhecimento, e a avaliação de sua qualidade é fundamental para a seleção de instrumentos que forneçam medidas válidas e confiáveis àquela população, respeitando suas particularidades^(23)^.

A validade de conteúdo refere-se ao julgamento sobre o instrumento, resulta do julgamento de diferentes examinadores especialistas, que analisam a representatividade dos itens em relação às áreas de conteúdo e a relevância dos objetivos a medir^(10)^. Nesse estudo, os juízes foram selecionados por desenvolverem pesquisas relacionadas à temática deste estudo, e convidados a participar espontaneamente.

Para analisar a validade de conteúdo, que se refere ao grau em que o conteúdo de um instrumento reflete adequadamente o construto que está sendo medido, utilizou-se o Índice de Validade de Conteúdo^(11)^. Três juízes analisaram cada questão atribuindo uma pontuação de 1 a 4, em que: 1 = item não claro; 2 = item pouco claro; 3 = item bastante claro; e 4 = item muito claro^(9)^. Foi contabilizada a contagem do número de questões com notas três e quatro, e total da contagem foi dividido pelo número total de questões avaliadas. O cálculo foi realizado para cada juiz individualmente, e para o total de juízes. O Índice de Validade de Conteúdo foi entre 0,94 e 0,97 para os juízes individuais, e de 0,96 para o total de juízes, o que demonstrou concordância aceitável entre os membros do comitê de especialistas, representando que os itens avaliados do questionário são medidas válidas e precisas^(11)^.

A validade de critério é a correlação existente entre a medida avaliada em relação à outra medida ou instrumento que serve como critério de avaliação, que possui atributos iguais ou semelhantes, e consiste na relação entre pontuações de um determinado instrumento e algum critério externo^(10)^.

No caso desse estudo, para a análise da validade de critério, os escores dos domínios de cada construto foram comparados entre dois grupos independentes constituídos a partir das respostas para questões que analisam o mesmo construto. Após a aplicação do questionário, foi realizada a testagem de três questões a fim de confirmar a validade de critério^(10)^. Para isso, os participantes foram orientados a responder “sim” ou “não” para as perguntas: a) “Você acredita que tem conhecimento adequado sobre os efeitos dos produtos agrotóxicos? “; b) “Você acredita que tem atitudes adequadas frente aos agrotóxicos?”, e c) “Você acredita que possui praticas seguras sobre os agrotóxicos?”.

Os resultados apontaram que não houve diferença ou associação entre as questões critério e os domínios ou perguntas do questionário. No caso do questionário CAP, não foi encontrado um questionário padrão-ouro para comparação. O questionário original em inglês não é validado, e por esse motivo não apresenta as análises para tal comparação.

É possível que o critério estabelecido, por apresentar perguntas diretas com respostas objetivas, não tenha favorecido, sendo um critério fraco de comparação, uma vez que limita as respostas das gestantes. Uma alternativa a ser pensada é criar uma escala que permita mais flexibilidade nas respostas. Ademais, também se acredita que isso tenha sido uma reflexão após ter respondido todas as questões do questionário e ter repensado acerca de seu conhecimento, atitudes e práticas.

Na literatura, se verifica situações semelhantes, como foi o caso da validação do “Protocolo de Avaliação do Frênulo Lingual em Bebês”, que na comparação da validade de critério, como não foi encontrado protocolos totalmente validados, considerados padrão-ouro na literatura, a análise da validade de critério, foi realizada por meio da comparação com o outro protocolo, que avaliava questões semelhantes^(24)^. A variação da força da correlação de média a forte encontrada para os aspectos considerados na presente pesquisa foi justificada pelas diferenças entre os itens e o modo de avaliação dos dois protocolos (Tabelas 1, 2, 3)^(25)^. Esses estudos apontam, de maneira geral, que há outras possibilidades de determinar a validade de critérios, indicando novas possibilidades para testar a validade nesse questionário.

Para constatar a validade do construto, são realizadas várias provas, que precisam ser analisadas em todos os seus detalhes. Esse tipo de validação visa a detectar, entre outros aspectos, quais as variáveis com as quais os escores do teste se correlacionam, quais os tipos de itens que integram o teste, o grau de estabilidade dos escores sob condições variadas e o grau de homogeneidade (se o teste mede um único traço ou se, ao contrário, mede traços diversos) do teste, com vistas a ter elementos que possam esclarecer o significado do instrumento^(10)^.

No caso deste estudo, a validade de construto é fundamental, uma vez que ele auxilia o pesquisador a determinar e entender melhor as questões cognitivas e psicológicas que estão sendo medidas pelo teste^(10)^. Ou seja, buscou-se verificar qual o conhecimento, atitudes e práticas de gestantes expostas a agrotóxicos sobre esses produtos. A validade de construto é subdividida em três tipos: teste de hipóteses, validade estrutural ou fatorial e validade transcultural^(9-11)^.

No teste de hipóteses, uma das estratégias dessa testagem é a técnica de grupos conhecidos, na qual grupos diferentes de indivíduos preenchem o instrumento de pesquisa e em seguida, os resultados dos grupos são comparados^(11)^. Nesse estudo, para essa análise, as gestantes do Grupo Expostas a Agrotóxicos foram comparadas as gestantes do Grupo Não Expostas a Agrotóxicos a fim de verificar diferenças nos escores dos domínios dos construtos de conhecimento, atitudes e práticas do Questionário CAP. A análise permitiu inferir que os grupos foram homogêneos para as variáveis idade, nacionalidade, estuda atualmente e renda familiar. A análise dos escores das perguntas dos construtos conhecimento, práticas e atitudes do Questionário CAP demonstrou que houve diferença estatística entre os grupos de gestantes, e apenas no domínio “Atitudes em casa” o Grupo Expostas a Agrotóxicos apresentou menor escore que o Grupo Não Expostas a Agrotóxicos. Isso identifica que as gestantes do Grupo Não Expostas a Agrotóxicos têm mais cuidados com a higienização de frutas e verduras antes de comer, com suas roupas de trabalho, e a própria casa em si. Sobre isso, ao se analisar questões referente a renda familiar e nível de escolaridade, que poderiam ser fatores que interferissem nessa questão, se observa que nesse estudo, essas variáveis são homogêneas.

Entretanto, o Grupo Expostas a Agrotóxicos apresentou maiores escores que o grupo Não Expostas nos domínios “conhecimento sobre população de risco”, “conhecimento dos sintomas de intoxicação”, “atitudes prejudiciais para o feto”, “responsabilidade para uso seguro dos agrotóxicos, ler rótulo das embalagens” “responsabilidade para uso seguro dos agrotóxicos, reutilização das embalagens”, “uso de agrotóxicos e cuidados”, “práticas de precaução”, “práticas referentes a animais domésticos”. É possível que, devido ao fato dos agrotóxicos não serem uma realidade próxima das gestantes não expostas, elas não tenham preocupação, ou conhecimento sobre o assunto, e talvez esses assuntos nunca tenham sido discutidos no ambiente em que vivem. Com as gestantes expostas, é o contrário; embora grande maioria delas não tenha tido instrução sobre o uso seguro e cuidado com os produtos, esse tema faz parte da vivência delas, e indiretamente está sendo comentado pelos vendedores de produtos, agrônomos, representante sindicais, e pelos próprios familiares, uma vez que há um senso comum e generalista de que o “veneno faz mal”.

Seguindo as etapas da validação do construto, a validade estrutural ou fatorial fornece ferramentas para avaliar as correlações em um grande número de variáveis, definindo os fatores, ou seja, as variáveis fortemente relacionadas entre si^(11)^. Nesse estudo, as características do instrumento não permitiram a análise fatorial, pois o Questionário CAP apresenta variação nos modelos de questões: possui questões com alternativas “sim” e “não”, com escala likert e dissertativa. Outra particularidade do questionário é que a resposta de uma questão, se sim ou não, interfere diretamente se irá responder a próxima questão ou seção do questionário. Essas singularidades dificultam a análise fatorial, uma vez que não consegue categorizar os dados.

Essa etapa foi realizada em estudo anterior^(7)^, que teve por objetivo traduzir, adaptar e desenvolver o estudo normativo preliminar do Questionário para a língua portuguesa. No referido estudo o questionário CAP^(6)^ foi traduzido para o português brasileiro, analisado por juízes de áreas relacionadas ao objeto do estudo, permitindo a revisão e adequação dos termos; na sequência foi aplicado em um grupo piloto, a fim de realizar a análise semântica e adaptação transcultural de termos. Após a adequação, o instrumento foi retrotraduzido para o inglês. A partir dos resultados foi possível verificar que esse instrumento foi coerente e satisfatório para o levantamento do conhecimento, atitudes e práticas de gestantes brasileiras em relação aos agrotóxicos. No presente estudo, verificou-se associações significativas entre o conhecimento e a fase de gestação, sendo que quanto mais tempo de gestação, maior foi o conhecimento sobre os riscos à exposição dos agrotóxicos, bem como a tomada de atitudes e práticas seguras durante o período gestacional, em casa e no trabalho.

Finalizando essa discussão, ressalta-se que neste estudo, foi realizada a validação do Questionário CAP em gestantes expostas aos agrotóxicos, por meio da validade de conteúdo, critério e construto. A pesquisa, desenvolvida em duas regionais de saúde do Estado do Paraná, não contou efetivamente com a totalidade de gestantes expostas, que expressariam a realidade cultural das regiões. Outra limitação é sobre as características do questionário, que por não seguir um padrão, não permitiu análises importantes, como a análise fatorial exploratória.

Por outro lado, o número significativo de gestantes que participaram do estudo, e o cumprimento de todas as etapas de confiabilidade e validade, permitiu a obtenção de dados confiáveis e a inferência de que o instrumento é válido para aplicação em populações brasileiras. Outro viés a ser destacado, é que apesar dos resultados favoráveis de validade, indicando a possibilidade de utilização deste Questionário CAP para levantamento do Conhecimento, das Atitudes e das Práticas de gestantes agricultoras no Brasil, a sua aplicação em forma de entrevista, com a presença das agentes comunitárias de saúde ou outro profissional da saúde pode ter gerado algum constrangimento, podendo resultar em alguma influência nas respostas, principalmente acerca de atitudes e práticas tomadas em casa.

Apesar de não ser confirmadas as evidências de que as práticas inseguras relacionadas aos agrotóxicos estejam associadas ao aumento da exposição da população brasileira a esses produtos, é possível que seja esse o principal motivo da ocorrência do aumento das alterações geradas por esses comportamentos de risco, no entanto, seriam necessárias medidas reais de exposição para confirmar essa hipótese. Esse não foi o foco deste estudo, mas as respostas obtidas a partir da aplicação deste instrumento podem ser úteis para futuras intervenções.

Pesquisas futuras devem ter como objetivo desenvolver instrumentos mais homogêneos e reduzidos, que facilitem a aplicação em um número ainda maior de sujeitos, e a análise completa da validação. Outro foco de interesse relacionado ao tema, seria a investigação dos homens, companheiros dessas gestantes, residentes do campo, quanto aos seus conhecimentos, atitudes e práticas frente aos produtos, no que diz respeito a preocupação com a gestação, uma vez que essa não deve ser uma preocupação única da mulher.

Os efeitos negativos dos agrotóxicos em gestantes e seus recém-nascidos merecem atenção das políticas públicas na área de saúde, pois conforme visto, pesquisas realizadas em vários países comprovam intercorrências na gravidez e na saúde do feto. As pesquisas científicas têm justamente a função de apresentar situações específicas para que, com medidas públicas eficazes, possam deixar de causar problemas à população.

Esse estudo pode ser um caminho inicial no entendimento da realidade das gestantes, e pode ser considerado um guia norteador de futuras práticas e ações voltadas ao cuidado da mulher exposta aos agrotóxicos. A validação das ferramentas de avaliação de conhecimentos, atitudes e práticas ajudará a desenvolver programas úteis e eficientes.

## CONCLUSÃO

Este estudo validou o questionário CAP considerando seu conteúdo, critério e construto. A análise desenvolvida indicou que as propriedades psicométricas da adaptação transcultural da versão brasileira da escala são consistentes e adequadas para aplicação no Brasil, o que permite a recomendação de aplicação do instrumento em contexto nacional. É importante que a versão validada do questionário seja aplicada em regiões do Brasil para entender as características culturais de cada região, a fim de propor medidas efetivas de promoção da saúde e prevenção de danos.
